# Transition-Metal-Oxide-Based Nanozymes for Antitumor Applications

**DOI:** 10.3390/ma17122896

**Published:** 2024-06-13

**Authors:** Huilin Sun, Yang Bai, Donghui Zhao, Jianhao Wang, Lin Qiu

**Affiliations:** School of Pharmacy, Changzhou University, Changzhou 213164, China

**Keywords:** nanozyme, transition metal oxide, antitumor, chemodynamic therapy

## Abstract

Transition metal oxide (TMO)-based nanozymes have appeared as hopeful tools for antitumor applications due to their unique catalytic properties and ability to modulate the tumor microenvironment (TME). The purpose of this review is to provide an overview of the latest progress made in the field of TMO-based nanozymes, focusing on their enzymatic activities and participating metal ions. These nanozymes exhibit catalase (CAT)-, peroxidase (POD)-, superoxide dismutase (SOD)-, oxidase (OXD)-, and glutathione oxidase (GSH-OXD)-like activities, enabling them to regulate reactive oxygen species (ROS) levels and glutathione (GSH) concentrations within the TME. Widely studied transition metals in TMO-based nanozymes include Fe, Mn, Cu, Ce, and the hybrid multimetallic oxides, which are also summarized. The review highlights several innovative nanozyme designs and their multifunctional capabilities. Despite the significant progress in TMO-based nanozymes, challenges such as long-term biosafety, targeting precision, catalytic mechanisms, and theoretical supports remain to be addressed, and these are also discussed. This review contributes to the summary and understanding of the rapid development of TMO-based nanozymes, which holds great promise for advancing nanomedicine and improving cancer treatment.

## 1. Introduction

Cancer continues to be a significant global public health issue and is currently the second most common cause of death worldwide [1]. Chemotherapy [2], radiotherapy [3,4], surgical resection, and immunotherapy [5] are the four most widely used methods in the treatment of malignant tumors. Although these methods can temporarily address cancer, their effectiveness is coupled with a reduction in the quality of life of cancer patients and a substantial increase in the cost of living. To address this problem and achieve better cancer treatment, many newly developed approaches have emerged in addition to the current main modalities of surgery, chemotherapy, and radiotherapy.

Photothermal therapy (PTT) [6,7], photodynamic therapy (PDT) [8,9,10], sonodynamic therapy (SDT) [11,12] and chemodynamic therapy (CDT) [13,14,15] have been gradually applied to anti-tumor treatment. The tumor microenvironment (TME) creates a favorable environment for tumor growth, proliferation, and metastasis due to factors such as mild acidity [2,16,17], adenosine triphosphate (ATP), the overproduction of H_2_O_2_ and antioxidants [18,19,20], hypoxia, and low catalase activity [21]. However, these factors also pose significant challenges to achieving selective and effective tumor treatments. For instance, tumor hypoxia can stimulate the activation of antioxidant enzymes and elevate intracellular redox glutathione (GSH) and antioxidant defense [21,22]. Consequently, it leads to a substantial decrease in the levels of reactive oxygen species (ROS), resulting in resistance to different therapeutic approaches, including ROS-related cancer therapy. Therefore, changing the tumor hypoxic environment, consuming excessive GSH, and increasing the ROS level in the tumor environment have become feasible approaches to tumor inhibition.

## 2. Nanozymes

Following a report by Gao et al. in 2007 regarding the peroxidase (POD)-like activity of Fe_3_O_4_ nanoparticles [23], the investigation of nanozymes as a group of nanomaterials with enzymatic properties has emerged as a rapidly growing field. Artificial enzymes have attracted more attention due to their unique advantages, such as good stability, high catalytic activity, and easy preparation/purification compared with natural enzymes [24,25,26,27]. Nanozymes are defined as “nanomaterials with enzyme-like properties”. Nanozymes have the potential to overcome the drawbacks of natural enzymes, such as instability, high expenses, and challenging storage conditions, and are gradually applied in many fields such as biomedical sensing, diagnosis and treatment, and environmental protection [24]. Compared with traditional anticancer drugs, nanomedicine mainly has the following advantages: improvement in the stability of the drug in vivo, thereby reducing the drug dosage and improving the therapeutic effect; precise therapy through active/passive targeting; reduction in the blocking effect of the physiological barrier and the prolongation of the circulation time of the drug in the body; and the facilitation of controlled drug release in vivo by constructing stimulus–response nanosystems, achieving multi-function and integrated diagnosis and treatment [28]. The unique properties of nanomaterials and the catalytic activity of natural enzymes endow nanozymes with the potential to participate in reactive oxygen species (ROS) generation and cancer therapy [29,30]. Transition-metal-based nanozymes were the first nanozymes to be identified and have attracted increasing interests as therapeutic candidates due to their excellent catalytic properties [31]. They can be classified as metal, metal oxide, metal-organic framework (MOF) [32,33], or hybrid nanozymes [34]. These transition-metal-based nanozymes have a variety of anti-tumor, anti-infective, and anti-inflammatory activities and are expected to be used as potential adjuvants, in adjuvant therapy, or as alternatives to cytotoxic chemotherapy drugs, antibiotics, and non-steroidal anti-inflammatory drugs [35,36,37].

**Figure 1:**
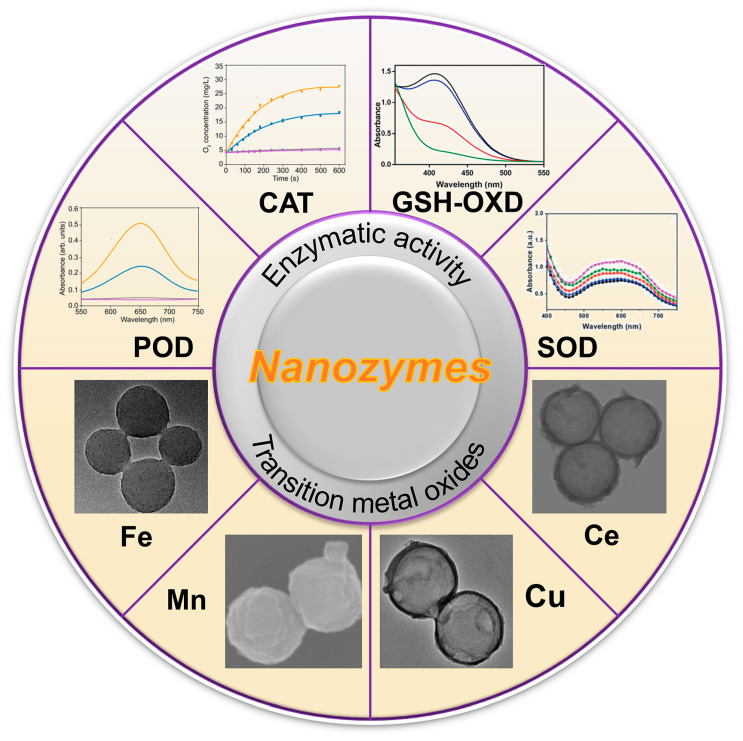
Summary of this review: enzymatic activities of TMO−based nanozymes and corresponding transition metal ions. POD: peroxidase, CAT: catalase, GSH−OXD: glutathione oxidase, SOD: superoxide dismutase.

Most nanozymes exhibit the properties of mimetic CAT or POD, which can either convert hydrogen peroxide (H_2_O_2_) into highly cytotoxic •OH to eliminate cancer cells or convert it into oxygen to enhance the hypoxic environment of tumors [38,39]. Unfortunately, the catalytic efficiency of these nanozymes is hindered by the highly intricate TME, which poses a significant challenge in achieving the intended therapeutic outcomes [40,41]. From this perspective, the intrinsic reactivity and specific regulatory systems of TME should be explored for effective anticancer therapies. Recently, multivalent metal nanozymes have been extensively explored to simultaneously enhance •OH production and reduce GSH overexpression [42,43]. The construction of multifunctional nanozymes is considered an ideal strategy to induce a variety of intratumoral responses that can be used for selective and efficient tumor therapy [44,45] (Figure 1).

## 3. Enzymatic Activities of Nanozymes

In recent years, nanozymes have been reported to exhibit a variety of enzymatic activities, which can decompose H_2_O_2_ into highly toxic •OH, or O_2_, or mimic glutathione peroxidase-like activity to consume glutathione in the TME, thus exerting anti-tumor effects [46,47,48,49]. The most frequently reported enzymatic activities are reviewed below.

### 3.1. Peroxidase (POD)-Mimicking Activity

**Figure 2:**
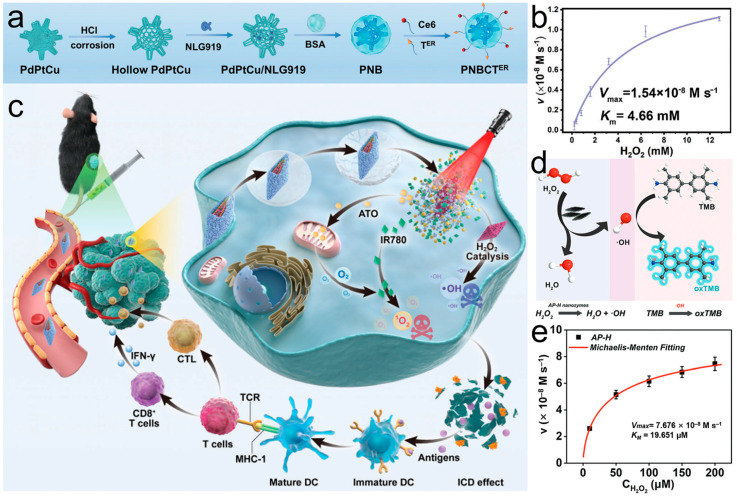
(**a**) Schematic illustration to indicate the synthetic procedure of hollow PBCTER nanozymes. (**b**) POD–mimicking–activity–related Michaelis–Menten kinetic analysis of PBCTER nanozymes [51]. (**c**) Therapy mechanism of AP-HAI nanoprobes in vivo. (**d**) Schematic demonstrating the peroxidase-mimicking activities of AP-H nanozymes. (**e**) Michaelis–Menten kinetic study of AP-H with H_2_O_2_ as substrate [52].

POD catalyzes the generation of highly cytotoxic hydroxyl radicals (•OH) from hydrogen peroxide (H2O2) in the TME, showing promise in inhibiting tumor growth [50]. The POD-like activity of nanozymes was detected using 3,3’,5,5’-tetramethylbenzidine (TMB), which was oxidized by the produced •OH. And a characteristic peak of oxTMB at 652 nm could be observed. Xie et al. [51] designed a multifunctional metal nanocomposite (PNBCTER) that integrates enzyme catalysis, photodynamic therapy (PDT), photothermal therapy (PTT), chemodynamic therapy (CDT), and immunotherapy for targeted cancer therapy (Figure 2a). The catalytic kinetics of the nanozyme were evaluated using a colorimetric reaction (TMB to oxTMB), and the K_m_ and V_max_ values were determined to be 4.66 mM and 1.54 × 10^−8^ Ms^−1^, respectively (Figure 2b). Zhang et al. [52] designed a nanozyme probe (AP-HAI) with high efficiency against lung cancer (Figure 2c). Enzymes, after modification by human serum albumin nanoparticles (AP-H), demonstrate excellent POD-like performance (Figure 2d). The K_m_ and the V_max_ values were 19.65 μM and 7.676 × 10^−8^ Ms^−1^ (Figure 2e). Zhu et al. [53] prepared a well-dispersed MnOOH nanocatalyst. It exhibits good POD enzymatic activity, thereby inhibiting tumor growth. MnOOH also exhibits CAT enzyme-like activity to catabolize H_2_O_2_ supplement O_2_ to regulate TME and catalyze GSH autooxidation.

### 3.2. Catalase (CAT)-Mimicking Activity

**Figure 3:**
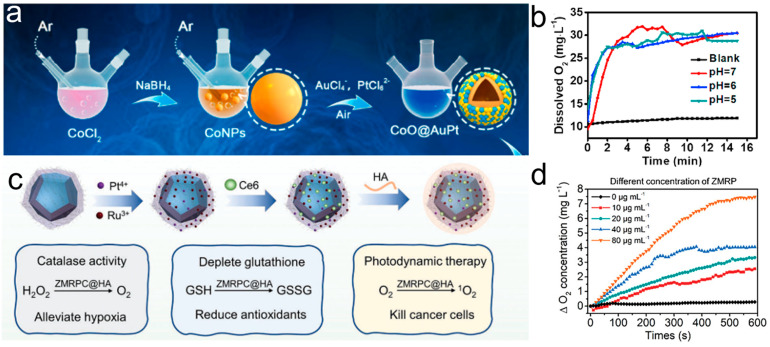
(**a**) Schematic illustration to indicate the synthetic procedure of hollow CoO@AuPt NPs. (**b**) A notable elevation of dissolved O_2_ levels was observed in less than 3 min, and the O_2_ generation rate exhibited an insignificant correlation with acidity [56]. (**c**) The fabrication process of ZMRPC@HA. (**d**) O_2_ generation after treatment with ZMRP of 0−80 μg/mL in the presence of 10 mm H_2_O_2_ [57].

CAT or CAT mimics have the ability to catalyze the decomposition of two H_2_O_2_ molecules to generate O_2_ and H_2_O, thereby preventing the accumulation of H_2_O_2_ and protecting organisms from oxidative damage caused by H_2_O_2_ [54,55]. Some nanomaterial-based nanozymes, such as transition metal oxide and multi-metal doped nanozymes, have been found to exhibit CAT-mimetic activity, causing H_2_O_2_ decomposition and continuous O_2_ production in tumor cells to regulate the hypoxic TME. Fu et al. [56] reported a novel CoO@AuPt nanocatalyst with multiple enzymatic activities, including POD, CAT, and oxidase (OXD) activities, to initiate intracellular hemodynamic responses in response to TME (Figure 3a). CAT-like enzyme activity was the most significant. The dissolved oxygen content was determined to rise from a concentration of 10 mg/L to 30 mg/L within 3 min, and the rate of oxygen generation was not significantly correlated with acidity (Figure 3b). Pan et al. [57] designed a bimetal ion-modified MOF nanozyme (Zr^4+^-MOF-Ru^3+^/Pt^4+^-Ce6@HA, ZMRPC@HA). ZMRPC@HA exhibits CAT-mimetic activity and can effectively regulate TME (Figure 3c). When the concentration of ZMRP was 80 μg/mL, the dissolved O_2_ in the solution significantly increased to 7.47 mg L^−1^ within 10 min compared with the control group (Figure 3d). In addition, ZMRPC@HA exhibits some POD enzymatic activity and glutathione oxidase (GSH-OXD)-mimetic activity, making it capable of producing toxic ROS and consuming GSH.

### 3.3. Glutathione Oxidase (GSH-OXD)-Mimicking Activity

**Figure 4:**
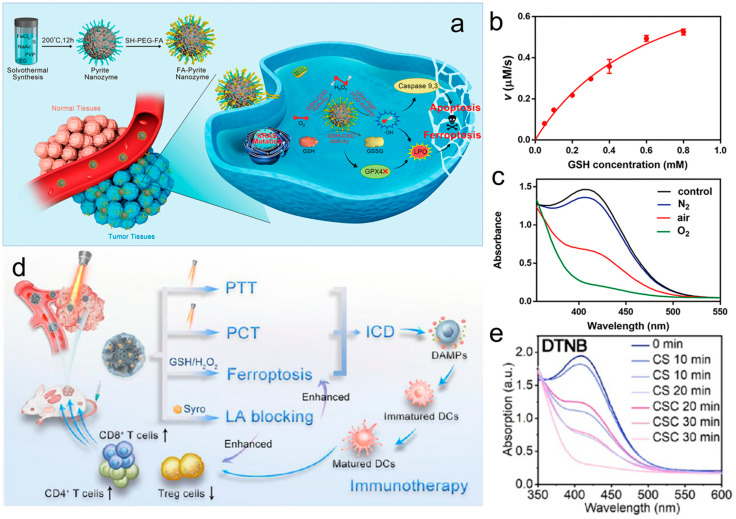
(**a**) Schematic illustration of the self-cascade pyrite nanozymes with ultrahigh POD-like catalytic activity and intrinsic GSH–OXD–mimicking ability for apoptosis−ferroptosis synergistic tumor therapy. (**b**) Kinetic assay for the GSH-OXD-like activity of pyrite nanozymes with GSH as substrate. (**c**) Reduction in GSH in an air, O_2_, and N_2_ atmosphere, respectively [59]. (**d**) The mechanism of potentiating antitumor immunotherapy induced by TME and NIR coactivatable synergistic PTT/PCT/Ferroptosis and improved by LA metabolic reprogramming of CSC@Syro. (**e**) GSH-OXD-like activity of CSC [60].

Glutathione, the major reducing small molecule in cells, exists in the form of free thiols, which can produce a highly reducing TME and protect cells from free-radical-induced oxidative damage. A widely used strategy to reduce intratumoral GSH levels is to convert the generated glutathione into glutathione disulfide (GSSG) through redox reactions [58]. Meng et al. [59] reported a high-performance pyrite nanozyme (FeS_2_) with glutathione-oxidase-like activity, which can oxidize glutathione to GSSG and H_2_O_2_ (Figure 4a). The pyrite nanozyme catalytic oxidation of reduced glutathione maximum velocity (V_max_) and Michaelis constant (K_m_) were 0.98 μMs^−1^ and 0.66 mM (Figure 4b). Moreover, the oxidation of GSH was further enhanced in an oxygen (O_2_) atmosphere and was inhibited in a nitrogen (N_2_) atmosphere (Figure 4c), indicating that O_2_ was involved in this reaction. Pyrite nanozymes also possessed POD-like enzyme activity. Yang et al. [60] designed a hollow mesoporous CuSe/CoSe_2_@syrosingopine (CSC@Syro) heterostructure as a multifunctional nanoadjuvant for the co-activation of TME and near infrared (NIR) waves (Figure 4d). Due to the presence of Cu^+^/Cu^2+^ and Co^2+^/Co^3+^ redox couplings, excess GSH can be consumed and further decompose endogenous H_2_O_2_ into •OH, resulting in potent ferroptosis. CSC has strong GSH-OXD activity, and 50 µg/mL CSC can completely consume 10 mM GSH in 30 min (Figure 4e).

### 3.4. Superoxide Dismutase (SOD)-Mimicking Activity

**Figure 5:**
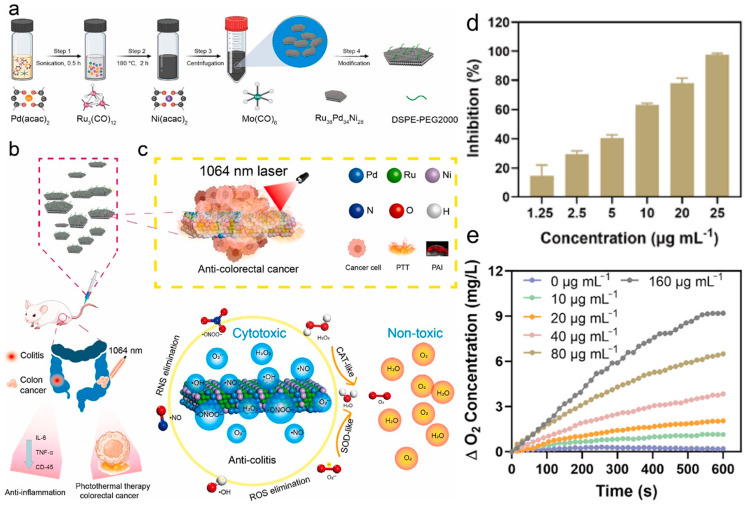
(**a**) Schematic illustration of the fabrication of TMNSs. (**b**,**c**) Scheme of TMNSs as multifunctional nanozyme−based nanoplatforms with RONS scavenging and photothermal conversion performance for colon disease treatment. (**d**) SOD−like activity of TMNSs. (**e**) O_2_ generation ability of TMNSs in the presence of H_2_O_2_ solution [61].

As a key antioxidant enzyme against ROS in cells, SOD catalyzes the dismutation reaction of O_2_^−•^ to generate O_2_ and H_2_O_2_. Therefore, SOD mimics can be used as a potential drug for the treatment of a variety of oxidative stress diseases [50]. 2-(4-iodophenyl)-3-(4-nitrophenyl)-5-(2,4-dithiophenyl)-2H-tetrazole) (WST-1), which can interact with O_2_^−•^, was used to study the ability of the nanozyme to consume O_2_^−•^, The formation of methazalazine showed a special absorption peak at 450 nm. Feng et al. [29]. reported a 2D vanadium carbide (V_2_C) MXene nanozyme with endogenous enzyme-like activity, such as SOD-enzyme-like activity, which can effectively catalyze O_2_^−•^ into H_2_O_2_ and O_2_. Notably, the amount of formazan was significantly reduced with increasing V_2_C MXenzyme concentration, with an inhibition efficiency of 50% at 200 μg/mL V_2_C MXenzyme. Meanwhile, V_2_C MXenzyme can also mimic five other naturally occurring enzymes, including CAT, POD, GSH-OXD, thiol peroxidase (TPx), and fluoroperoxidase (HPO). Wang et al. [61] created Ru_38_Pd_34_Ni_28_ ultrathin trimetal nanosheets (TMNSs) by incorporating various transition metal atoms into the RuPd nanosheet structure (Figure 5a). TMNSs possess significant CAT- and SOD-like nanozyme activities (Figure 5b,c). The study found that the amount of O_2_ generated was directly proportional to the concentration of both TMNSs and H_2_O_2_, indicating the exceptional CAT-like activity of TMNSs in effectively removing H_2_O_2_ (Figure 5e). Moreover, when the concentration was 25 μg/mL, the inhibition rate was close to 100% (Figure 5d). Under normal physiological conditions, cerium oxide nanozymes have properties similar to SOD and CAT, which have been explored as antioxidants to alleviate a wide range of types of oxidative stress [62]. Gao et al. [63] developed a pH-responsive “oxidative cycle accelerator” using a black phosphorus/cerium catalytic nanozyme (BP@CeO^2^-PEG) that can be tuned to alleviate acute kidney injury (AKI) induced by platinum compounds (DDP). The nanozyme promotes ATP synthesis and increases the Ce^3+^/Ce^4+^ ratio to effectively scavenge ROS in a sustained manner. The BP@CeO_2_-PEG nanozymes exhibited remarkable SOD-like activity, as evidenced by an inhibition rate of almost 40% at a concentration of 80 μg/mL. BP@CeO_2_-PEG also has CAT and hydroxyl radical antioxidant capacity (HORAC).

## 4. Transition Metal Oxide Nanozymes

Transition metal oxides (TMOs) have emerged as one of the most extensively studied classes of transition-metal-based nanozymes due to their unique properties and diverse applications. TMOs exhibit diverse catalytic activities, including OXD-like, POD-like, and SOD-like activities, making them promising candidates for various applications in biomedicine, biosensing, and environmental remediation. The unique physicochemical properties of TMOs, such as their morphology, tunable composition, and surface chemistry, allow for precise control over their catalytic performance. Additionally, TMOs possess inherent stability and durability, making them suitable for practical applications under harsh conditions. Their catalytic mechanisms involve the redox reactions of transition metal ions and the generation of ROS, which facilitate the conversion of substrates into desired products. The development of TMO-based nanozymes, including Fe, Mn, Cu, Ce, and hybrid metals, are reviewed.

### 4.1. Iron

**Figure 6:**
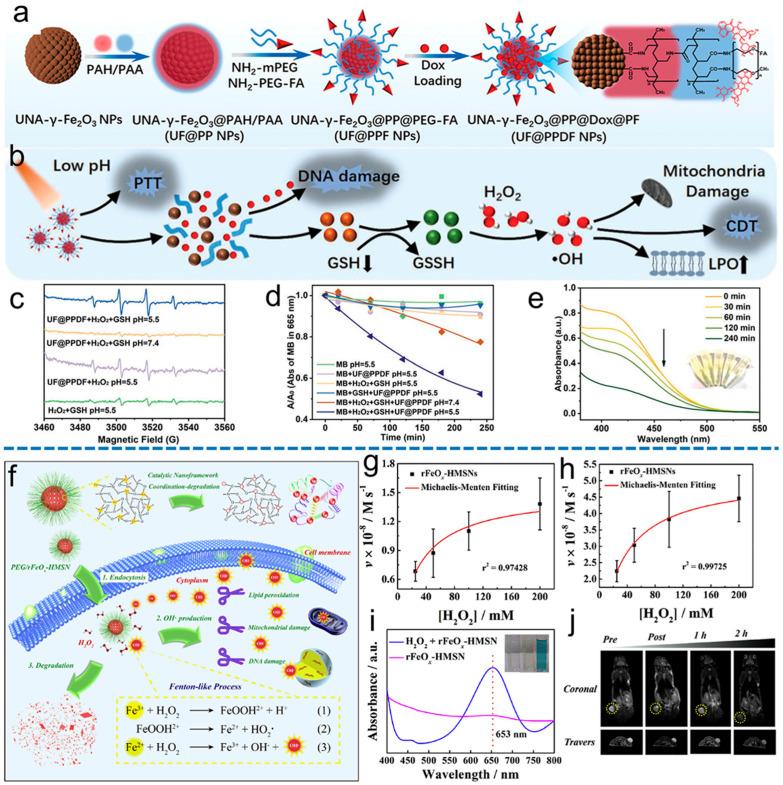
(**a**) Production of UF@PPDF NPs with assembled ultra–small γ–Fe_2_O_3_ nanocrystals, loaded with the chemotherapeutic drug Dox and cancer cell targeting molecules. (**b**) The mechanism of the UF@PPDF NPs for PTT–chemotherapy–ferroptosis trifunctional synergistic cancer therapy. (**c**) EPR spectra demonstrating •OH generation by UF@PPDF NPs, with 5,5-dimethyl-1-pyrroline-Noxide (DMPO) serving as the •OH trapping agent. (**d**) Changes in UV-Vis spectra showing MB degradation in different systems. (**e**) Time–dependent GSH depletion by UF@PPDF NPs, with DTNB as the trapping agent [66]. (**f**) A visual representation of the therapeutic effectiveness of rFeO_x_–HMSN nanocatalysts. An analysis of the relationship between the initial velocity of hydroxyl radical generation and H_2_O_2_ concentration by using Michaelis–Menten fitting curves, with TMB concentrations of 0.8 mM and 1.6 mM, is shown in (**g**,**h**), respectively. (**i**) The absorbance of TMB aqueous solution under acidic conditions (pH = 6.0) with the addition of rFeO_x_–HMSN and H_2_O_2_, as well as H_2_O_2_ alone. (**j**) The in vivo *T*_2_–weighted MR imaging of mice bearing 4T1 tumors before and after the intravenous administration of PEG/rFeO_x_–HMSN nanocatalyst over a prolonged period of time [67].

Fe_3_O_4_ nanoparticles (NPs) were the first to be reported to possess POD-like activity, which proved that Fe_3_O_4_ NPs are strong candidates for the preparation of nanozymes. Inspired by this pioneering work, POD mimics have been extensively explored and studied, such as Fe_3_O_4_ (magnetite), Fe_2_O_3_ (hematite), and doped ferrites [64]. Iron-based nanomaterials can also be used as *T*_2_-weighted MRI contrast agents [65]. Qin et al. [66] developed UF@PPDF NPs, which are multilayer Fe_2_O_3_ structures created by modifying an ultra-small γ-Fe_2_O_3_ nanocrystal assembly with folic acid (FA) targeting groups and the chemotherapeutic drug doxorubicin (Figure 6a). The production of •OH was verified using electron paramagnetic resonance (EPR) spectroscopy, with 5, 5-dimethyl-1-pyrroline-N-oxide (DMPO) utilized as the •OH-trapping agent (Figure 6c). Additionally, a lighter color of methylene blue was observed, providing further evidence of •OH generation (Figure 6d). The involvement of GSH was verified by observing a gradual decrease in the absorption of the GSH probe 5,5’-dithiobis-(2-nitrobenzoic acid) (DTNB) over time (Figure 6e). These effects induce ferroptosis in tumor cells in a synergistic manner (Figure 6b). Wang et al. [67] reported an iron engineering framework for mesoporous silica nanoparticles (MSNs) to create a biodegradable and catalytic nanocatalyst (rFeO_x_-HMSN) with a superferromagnetic framework and *T*_2_-MRI properties via a “dissolving regeneration” strategy (Figure 6f). rFeO_x_-HMSN nanocatalysts can trigger the Fenton reaction in situ, which generates highly toxic hydroxyl radicals to kill cancer cells (Figure 6i). The Lineweaver–Burke plot was utilized to determine the Menten constant K_m_ and maximum velocity V_max_, resulting in K_m_ values of 29.57 mM and 31.43 mM for rFeO_x_-HMSN nanocatalysts when exposed to 0.8 mM and 1.6 mM TMB, respectively. The rFeO_x_-HMSN nanocatalyst displayed a maximum •OH radical production rate of 14.272 nM s^−1^ when exposed to 0.8 mM TMB, while this rate increased to 49.312 nM s^−1^ when exposed to 1.6 mM TMB (Figure 6g,h). The PEG/rFeO_x_-HMSN nanocatalyst exhibited a negative MR imaging performance and was observed to accumulate within the tumor tissue. This led to a notable reduction in the *T*_2_-weighted negative MRI signal in the tumor tissue. This effect is likely due to the enhanced permeability and retention (EPR) effect, as demonstrated by the circled region in the MR images (Figure 6j). Mao et al. [68] designed and constructed a smart nano-catalytic platform, the DMSN-Au-Fe_3_O_4_ NP, which for the first time took advantage of the unique catalytic activity of Au NPs co-modified on dendritic mesoporous silica NPs mimicking glucose oxidase (GOx) and the POD of Fe_3_O_4_ NPs. It catalyzed the TME response cascade and promoted the production of H_2_O_2_ and ROS.

### 4.2. Manganese

**Figure 7:**
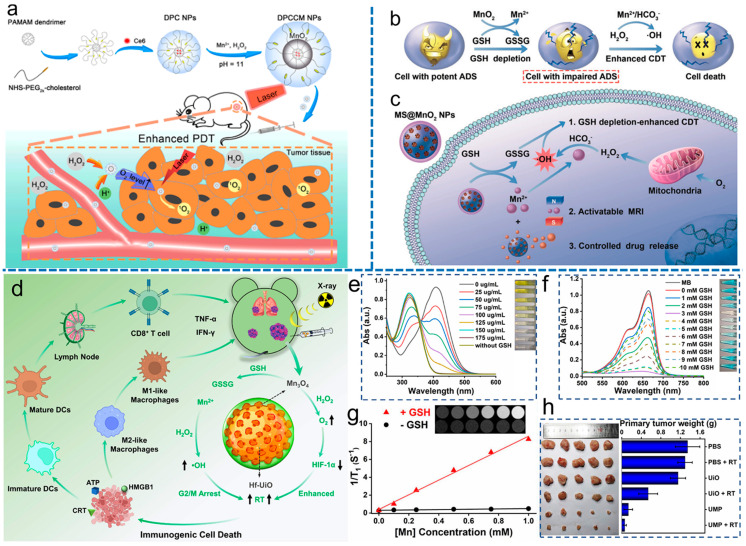
(**a**) Synthesis of DPCCM NPs and their application in pH/H_2_O_2_–responsive enhanced photodynamic therapy [76]. (**b**) The mechanism of MnO_2_ as a smart chemodynamic agent for enhanced CDT for cancer. (**c**) Illustration of the application of MS@MnO_2_ NPs for MRI-monitored chemodynamic combination therapy [70]. (**d**) Illustration of multiple strategies for immunogenic radiotherapy for UMP. (**e**) The absorption spectra of DTNB in the UV–Vis range after its degradation by GSH treated with varying concentrations of UM. Inset: the corresponding photo. (**f**) The absorption spectra of MB in the UV–Vis range after degradation by H_2_O_2_ and GSH-treated UM. Inset: the corresponding photo. (**g**) *T*_1_-weighted MR images of UM solution containing varying concentrations of Mn (0, 0.1, 0.25, 0.5, 0.75, and 1 mM) with or without 10 mM GSH treatment. (**h**) Images of the primary tumors and their corresponding weights obtained from different groups on the 16th day [79].

Compared with Cr, Co, and other elements with high biological toxicity, Mn is a natural and non-toxic element, making it a more appropriate choice for use in biological applications [69]. Manganese-based nanomaterials, including manganese dioxide (MnO_2_) and manganese tetroxide (Mn_3_O_4_), have the advantages of better response to TME, wider pH range [70], and closer radius to Fe. It can also use its own Fenton effect to catalyze H_2_O_2_ to produce toxic •OH, which can be used for anti-tumor applications [71]. MnO_2_ has the ability to act as a catalyst for the decomposition of hydrogen peroxide (H_2_O_2_) to generate oxygen. Due to its properties as an inorganic nanozyme, MnO_2_ has significant potential for use in multifunctional nanomaterials designed for cancer therapy [72,73]. In addition, MnO_2_ reacts with GSH to form Mn^2+^, which can be used for magnetic resonance imaging (MRI) [74,75]. Xu et al. [76] developed a nanoplatform called the Ce6/MnO_2_@DPC NP (DPCCM NP) that is responsive to changes in pH and the presence of hydrogen peroxide (H_2_O_2_). This nanoplatform exhibits exceptional CAT enzyme activity and possesses various functions that enhance cellular uptake, stimulate oxygen generation, and improve the efficacy of PDT (Figure 7a). Lin et al. [70] reported a self-strengthening CDT nanoagent (MS@MnO_2_), which has the ability to deliver Mn^2+^ in a Fenton-like manner and deplete glutathione (GSH). This nanoagent acts as an intelligent chemokinetic agent that disrupts cellular antioxidant defense systems (ADS) and supplies cells with •OH producers, ultimately resulting in enhanced cancer cell death through CDT (Figure 7b,c). Due to the presence of Mn^2+^, MS@MnO_2_ can be used for combined tumor therapy under magnetic resonance imaging (MRI) monitoring.

Compared to copper zinc superoxide dismutase (Cu/Zn SOD) and iron superoxide dismutase (Fe SOD), natural manganese superoxide dismutase (Mn SOD) has shown superior efficacy in treating chronic diseases. This has motivated researchers to work on synthesizing SOD based on manganese, with the goal of developing more effective treatments for these conditions [77]. Yao et al. [78] demonstrated that Mn_3_O_4_ NPs synthesized by hydrothermal method had significant SOD-like enzyme activities and CAT-like enzyme activities as well as hydroxyl radical scavenging activity due to the dual oxidation state of Mn^2+^ and Mn^3+^. At a concentration of 20 mg/mL of Mn_3_O_4_ NPs, the removal efficiency of O_2_^−•^ was approximately 75%, and that of H_2_O_2_ was about 75%, which was even more efficient than that of 10 U mL^−1^ CAT. Huang et al. [79] synthesized UiO-66(Hf)-NH_2_ and then coated Mn_3_O_4_ particles in it, resulting in a core–shell structure named UiO@Mn_3_O_4_ (UM) (Figure 7d). The gradual disappearance of the yellow color of TNB with increases in the concentration of UM, as depicted in Figure 7e, suggests that UM fully consumed GSH. Subsequently, the Mn^2+^-mediated Fenton-like reaction was investigated (Figure 7f). In Figure 7g, it can be observed that UM treated with GSH displayed paramagnetic characteristics, with an r_1_ value of 8.20 mM^−1^ s^−1^. Additionally, the *T*_1_-field MRI signal increased gradually as the concentration of UM increased. The UiO@Mn_3_O_4_@PAA (UMP) nanocomposite was found to be an effective nanoregulator for relieving hypoxia and inducing oxidative stress through the Fenton-like reaction, which was enhanced by GSH depletion. UMP exhibited excellent efficacy in eliminating primary, distant, and metastatic tumors (Figure 7h).

### 4.3. Copper

**Figure 8:**
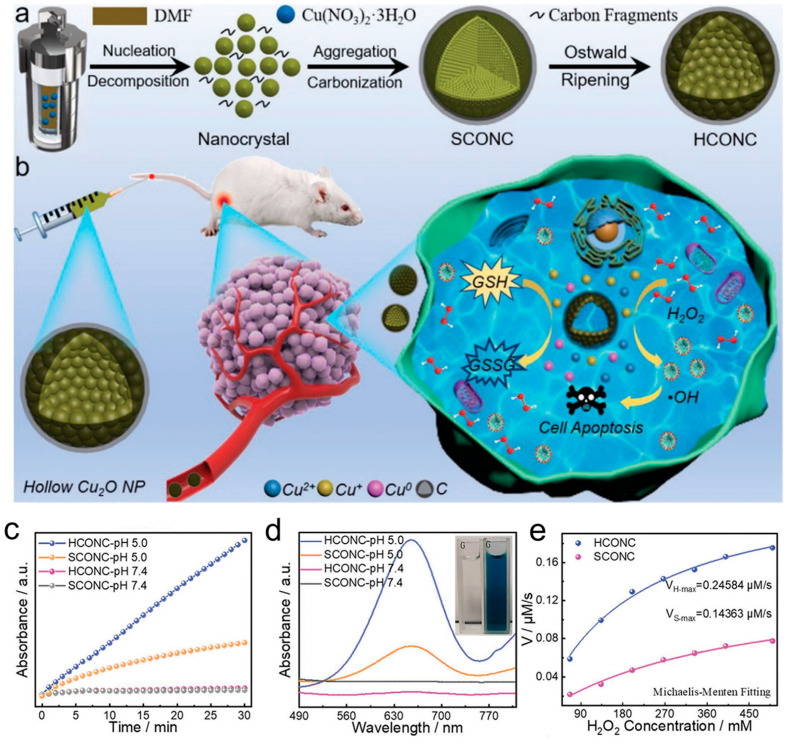
(**a**) The formation process of HCONC. (**b**) HCONC-catalyzed cascade reaction for CDT. (**c**,**d**) The catalytic kinetics of HCONC and SCONC were studied under neutral and acidic conditions at room temperature, and their steady–state behavior was noted. (**e**) The relationship between the activity of •OH generation and the concentration of H_2_O_2_ in the presence of HCONC or SCONC was analyzed using Michaelis–Menten fitting curves [88].

Copper-based nanomaterials, such as copper nanoparticles (Cu NPs), copper sulfide (CuS), copper selenide (Cu_2_Se), copper oxide (CuO), etc., have high photothermal conversion rates, and they can catalyze H_2_O_2_ in TME to produce excess •OH, which reshapes the TME and induces the death of tumor cells [80,81]. Cuprous oxide (Cu_2_O), as a stable copper-based nanomedicine, has been confirmed to promote tumor cell apoptosis by producing ROS in melanoma, bladder cancer, and cervical cancer cell lines [82,83]. Wang et al. [84]. constructed a bifunctional nanocatalyst called Cu_2_O@Dex, which was composed of dextran. This nanocatalyst has the ability to restore normal tumor perfusion and oxygenation in a straightforward manner. Through pH-sensitive dual catalytic functions, Cu_2_O@Dex generates both nitric oxide (NO) and •OH simultaneously. Under weakly acidic conditions (pH 6.5), the release of Cu (Cu^+^/^2+^) from Cu_2_O@Dex initiates the catalytic process, leading to the production of NO and subsequent vascular normalization. In addition, Cu_2_O@Dex further released a mass of Cu (Cu^+^/^2+^) under more acidic intratumoral cells (pH 5.5), which rapidly catalyzed •OH generation and enhanced CDT efficacy. Since copper is an essential trace element in the human body and is involved in the functioning of various enzymes, including tyrosinase and Cu-Zn SOD [85,86], it is logical to assume that copper-based nanomaterials can be utilized to eliminate ROS. Liu and colleagues [87] demonstrated the straightforward and effective one-step synthesis of ultra-small Cu_5.4_O NPs (Cu_5.4_O USNPs) that exhibit diverse enzyme-like properties and a wide range of ROS scavenging capabilities for the treatment of ROS-related diseases. These Cu_5.4_O USNPs not only function as CAT, SOD, and GSH-OXD mimics but also showed protective effects against ROS-induced damage at very low concentrations, leading to significantly improved therapeutic outcomes in conditions such as acute liver injury, acute kidney injury, and wound healing. Meng et al. [88] created dual-shell structures consisting of hollow cuprous oxide and nitrogen-doped carbon (HCONC) (Figure 8a). These structures were specifically designed to serve as nanozymes for oncotherapy through CDT (Figure 8a,b). The Fenton-like reaction mediated by Cu^+^ can effectively break down H_2_O_2_ to produce •OH under relatively mild conditions. In comparison to SCONC, the colorimetric reaction and the ability to generate •OH between TMB and HCONC were more pronounced and rapid (Figure 8c,d). Both SCONC and HCONC showed Michaelis–Menten and Lineweaver–Burke plots, but the •OH generation activity of HCONC was significantly greater than that of SCONC (Figure 8e).

### 4.4. Cerium

**Figure 9:**
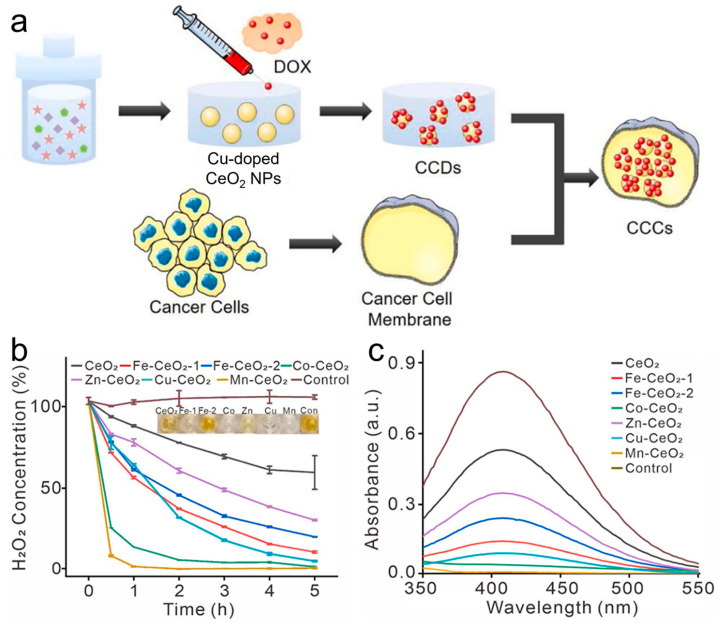
(**a**) Schematic illustration of CCC NPs for synergistic cancer therapy. (**b**) Quantitative analysis of the residue H_2_O_2_ in the solution after the application of M–CeO_2_ NPs under physiological pH conditions. (**c**) The absorption spectra of the H_2_O_2_ indicator [Ti(SO_4_)_2_] solution [95].

The exceptional catalytic activity of CeO_2_ nanozymes has led to significant advancements in cancer treatment [89,90]. The rapid conversion between Ce^3+^ and Ce^4+^ results in the generation of oxygen vacancies on the surface of CeO_2_, which determines the POD and OXD activities of CeO_2_ [91,92,93]. Cheng et al. [94] designed a stable nanocomposite material (CeO_2_/Y) modified by highly dispersed CeO_2_ nanoparticles on Y-type zeolite as a carrier and synthesized the CeO_2_/Y nanocomposite material through a simple wet impregnation method. CeO_2_/Y nanocomposites were also proposed for the first time as an efficient POD-mimicking nanozyme for the accurate detection of H_2_O_2_ and glucose. Using this POD-like activity, Cheng and colleagues [95] constructed a novel anti-tumor controlled release system (Cu-CeO_2_ NPs) loaded with breast cancer cell membrane and the clinical anticancer drug DOX for cancer treatment (Figure 9a). The addition of copper ions to the CeO_2_ nanozymes resulted in a significant increase in the Ce^3+^/Ce^4+^ ratio, leading to a significant improvement in the POD-like activity of the TME-specific cancer therapy drug. To test the ROS scavenging ability of CeO_2_ NPs under physiological conditions, H_2_O_2_ was used as the most prevalent type of ROS. The complete removal of H_2_O_2_ was observed within 5 h of the introduction of Co-CeO_2_, Mn-CeO_2_, and Cu-CeO_2_ NPs, indicating a significantly higher catalytic efficiency compared to other types of CeO_2_-based nanoparticles (Figure 9b,c). Through the implementation of site-selective growth and steric restriction strategies, Ma et al. [96] developed a distinctive pushpin-shaped Au/CeO_2_ hybrid nanozyme that exhibits exceptional catalytic activity. Au/CeO_2_ has superior catalytic activity and targeting ability and exhibits a good anti-tumor effect in vitro and in vivo.

### 4.5. Multimetallic Oxide

**Figure 10:**
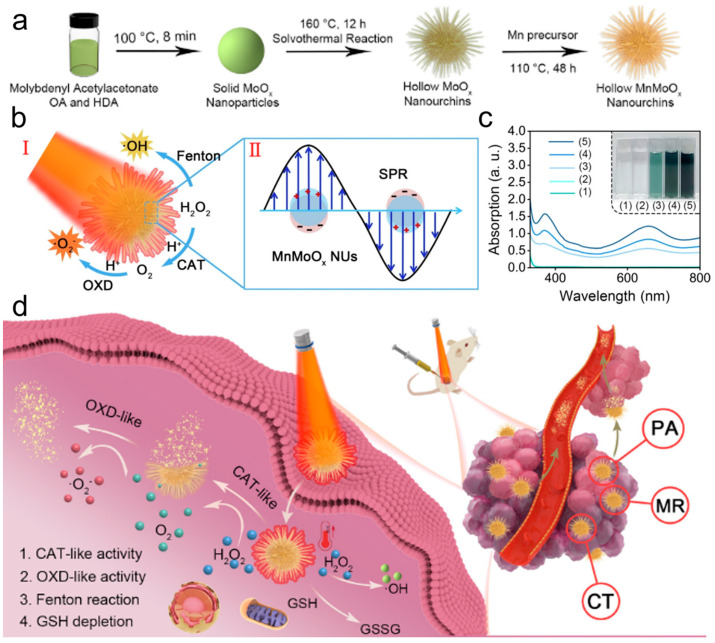
(**a**) Schematic diagram of the synthetic process of the MnMoO_x_ NUs. (**b**) Scheme of the catalytic process (**I**) and enhanced SPR (**II**) of MnMoO_x_ NUs. (**c**) The absorption spectra of TMB and corresponding digital photos of different groups. (**d**) Illustration of the working mechanisms of the MnMoO_x_ NUs [104].

Recently, the distinctive biological, physical, and chemical properties of transition-metal-based nanomaterials upon entering tumor cells, known as biological effects, have garnered significant attention [97]. The significance of metal ions and metal-based nanomaterials in cancer treatment cannot be overstated, as demonstrated by the various roles they play. For instance, Ca^2+^ can regulate T cell receptor activation, K^+^ can control stem cell differentiation, Mn^2+^ can activate the STING pathway, and Fe^2+^/^3+^ can facilitate tumor ferroptosis and augment catalytic therapy [98]. However, the therapeutic effect of a single metal material is limited. Therefore, a growing number of metal composite materials have been designed and applied [99,100]. The cGAS-STING pathway can be activated by Mn^2+^ and Zn^2+^, which implies that they possess the capacity to function as immune activators [101,102]. Lei et al. [103] designed the bimetal oxide manganese molybdate nanoparticle MnMoO_x_-PEG (MMP NDs), which was constructed from Mn^2+^ and MoO_4_^2−^ and modified by distearoyl phosphatidylethanolamine-polyethylene glycol 5000 (DSPE-PEG_5k_). The presence of high-valence Mo^6+^ and Mn^4+^ in MMP NDs allows for their reduction into a low-valence Mo^5+^ and Mn^2+^ by GSH. This is believed to enhance their ability to facilitate ferroptosis. When the incubation time was prolonged, the characteristic UV-Vis absorption peak of DTNB at ~412 nm exhibited a marked decrease, indicating the efficient GSH consumption capacity of MMP NDs. MMP NDs, when administered intravenously, can effectively reverse the immunosuppressive tumor microenvironment (TME), while also facilitating both the initiation and enhancement of cancer immunotherapy (CIT), without requiring any additional immune adjuvants. Zhang et al. [104] introduced a composite nanoenzyme (MnMoO_x_), obtained by thermally injecting manganese into the molybdenum oxide semiconductor (Figure 10a). The cascade CAT and OXD activity (Figure 10b) were directly influenced by the surface plasmon resonance (SPR) effect. The production of ROS was indirectly confirmed through the UV-Vis absorption spectra and digital photographs of the different groups using TMB as probes, as shown in Figure 10c. The composite was utilized to perform antitumor therapy, which was guided by tri-modal imaging and activated by the tumor microenvironment, while also involving a cascade catalytic process (Figure 10d).

**Figure 11:**
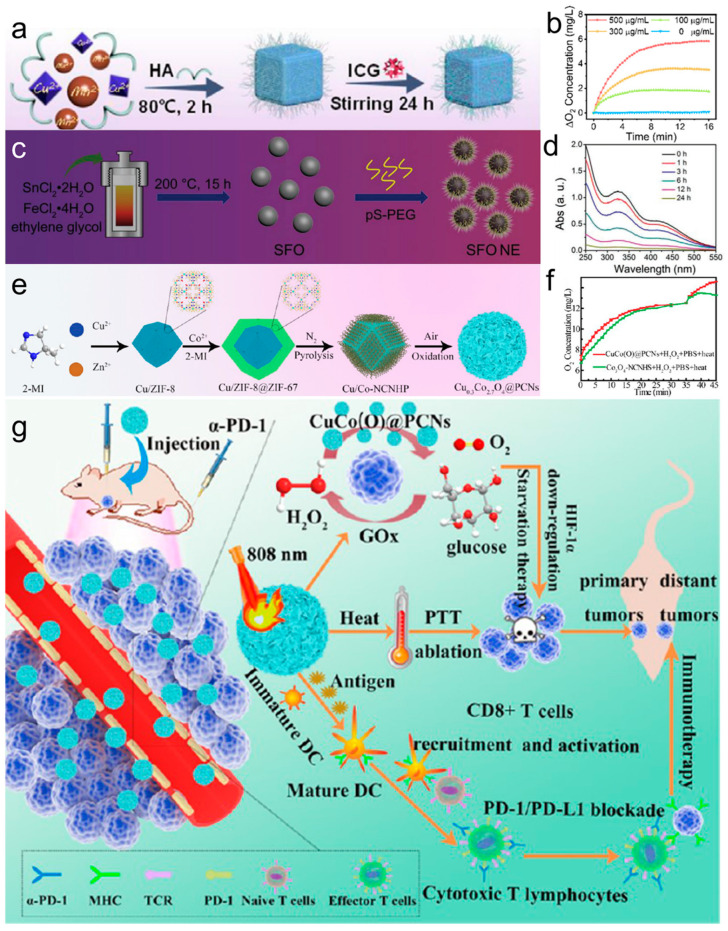
(**a**) Schematic diagram of the synthetic process of the CMOI NCs. (**b**) O_2_ generation curves of CMOI NCs aqueous solutions with different concentrations [106]. (**c**) Schematic illustration for the synthetic process of SFO. (**d**) Time-dependent GSH depletion by SFO [111]. (**e**) Fabrication procedure of the CuCo(O)@PCNs nanoenzyme. (**f**) Comparison of O_2_ generation of the Co_3_O_4_-NCNHS and CuCo(O)@PCNs samples. (**g**) Schematic illustration of the ZIF-derived nanozyme CuCo(O)@PCNs with three-in-one functions to achieve synergetic therapy [112].

Liu et al. [105] introduced a new biomimetic platform (ZnMnFe_2_O_4_–PEG–FA) with both photothermal and catalytic activity for tumor therapy. This platform exhibits excellent photothermal effects, with a photothermal conversion efficiency (*η*) of approximately 47.8%. Moreover, it demonstrates remarkable POD-like activity, with determined K_m_ and V_max_ values of 45.2 mM and 1.62 × 10^−7^ Ms^−1^, respectively. The combination of these properties enables synergistic tumor cell diagnosis and ablation. Lv et al. [106] developed a multinanozyme system, known as HA-CuMnO_x_@ICG nanocomposites (CMOI NCs), by incorporating indocyanine green (ICG) into hyaluronic acid (HA)-stabilized CuMnO_x_ nanoparticles (CMOH) via a straightforward synthesis method (Figure 11a). The CMOI NCs demonstrated enhanced multienzyme catalytic activities, including POD-like, CAT-like (Figure 11b), and OXD-like activities, along with the ability to deplete GSH. These properties, combined with photothermal enhancement, enabled synergistic photothermal therapy and enhanced tumor oxidation.

The presence of multivalent metal ions (Sn^2+^/Sn^4+^ and Fe^2+^/Fe^3+^) and strong absorption in the NIR region make bimetallic oxides, such as tin ferrite (SnFe_2_O_4_, referred to as SFO), highly appealing in the field of photocatalysis. As a result, SFO has garnered significant attentions [107,108,109,110]. Feng et al. [111] proposed a novel TME-regulated nanozyme using tin ferritate (SnFe_2_O_4_) for simultaneous PTT, PDT, and CDT (Figure 11c). The presence of Sn^2+^/Sn^4+^ and Fe^2+^/Fe^3+^ redox coupling enables SFO to exhibit excellent •OH generation capacity through Fenton-like reactions, while eliminating overexpressed GSH in TME and reducing tumor antioxidant capacity through GSH-OXD-like activity (Figure 11d). Importantly, SFO nanozymes can interact with endogenous H_2_O_2_ to produce O_2_, thereby alleviating hypoxia in the TME. Wang et al. [112] synthesized Cu-Co oxide porous carbon nanocomposites (CuCo(O)/GOx@PCNs) loaded with GOx via pyrolysis and the calcination of Cu-doped bilayer MOF (Figure 11e). The hybrid nanozyme system was able to achieve three distinct functions, which included oxygen generation (Figure 11f), glucose consumption, and photothermal conversion, all within a single system. CuCo(O)/GOx@PCNs can enter tumor cells to regulate TME and alleviate hypoxia, achieving the synergistic treatment of starvation therapy and PTT and inducing a systemic immune response to inhibit the growth of distant tumors (Figure 11g). The representative nanozymes with multienzymatic activities for enhancing tumor therapy are selected and listed in Table 1.

## 5. Improvement Strategies and Technologies of Nanozymes

To enhance the potential of nanozymes as substitutes for natural enzymes, researchers must focus on optimizing their specificity and catalytic efficiency through innovative design strategies and advanced fabrication techniques. The activities of nanozymes can be greatly modulated via the regulation of morphology, valence, composition, the architecture of the active sites, and surface modification, which have been universally proven recently. Smaller size or propriate morphology can expose more active sites and elevate catalytic activity of nanozymes by increasing surface-to-volume ratio and facilitating mass transport [113,114,115,116,117]. Surficial coatings of nanozymes via the physisorption of small molecules, ions, and polymers or covalent bonding contribute to their activity adjustment by causing variations in surficial charge and microenvironment [118]. The surface modification of nanomaterials not only acts as a stabilizer in the synthesis of nanomaterials, but also provides a reaction site for the further coupling of functional groups. The catalytic performance is also heavily influenced by the surface valence conditions and the presence of oxygen vacancies within its structure. To enhance the catalytic efficiency of the nanozyme, it is crucial to optimize its structural composition through strategies such as alloying or doping. New strategies have also been proposed to elevate catalytic activity by mimicking the nanostructure of the active sites or the enzymatic microenvironment of natural enzymes.

Besides experimental technique development, computational protocols involving the development of new atomistic force field parameters, flexible docking with Brownian dynamics, and µs-long MD simulations contribute greatly to the microscopic view at the basis of experimental results for nanozymes [119,120]. The computational methodology is beneficial for problem solving, such as tuning the catalytically active sites, as well as designing proper nanostructures with target enzymatic activity in nanomedicine in the future [121,122].

Despite the significant progress made, several obstacles still hinder the further advancement and practical implementation of nanozymes. The large-scale production of nanozymes is easily influenced by reaction conditions, and their catalytic activity and selectivity still need enhancement. While various synthesis methods have been reported, there has been no groundbreaking innovation compared to traditional nanomaterial fabrication techniques. Although some new mechanisms have been reported in the study of nanozymes’ catalytic properties, the precise 3D structure of their active sites remains unknown. However, these challenges can be overcome through persistent efforts and continuous advancements in material science, chemistry, nanoscience, and technology. In addition to experimental work, researchers should also focus on theoretical calculations, which can help reveal new catalytic mechanisms of nanozymes. By combining experimental and computational approaches, we can deepen our understanding of nanozymes toward their widespread use.

## 6. Limitations

Despite significant progress in the application of TMO-based nanozymes for tumor therapy, several challenges remain to be addressed, which are listed as follows.

(1) Although many multifunctional nanozymes have demonstrated promising results in vitro, their potential for clinical applications remains uncertain. For example, nanozymes should be designed to be hypersensitive to H_2_O_2_ for degradation since the concentration of H_2_O_2_ in vivo is around a few micromoles.
(2) The cytotoxicity and biocompatibility of TMO-based nanozymes has not been fully confirmed, especially for nanomaterials containing transition metals. Further studies are required to investigate the mechanisms and pharmacokinetics of nanozymes based on TMOs before they can be considered for clinical application.
(3) Nanozymes possessing multiple enzymatic activities have the ability to catalyze a diverse array of substrates. The multifunctional properties of nanozymes could be beneficial for expanding their applications in tumor therapy; however, they may also introduce some adverse factors. It is crucial to enhance the targeting ability of nanozymes and validate their localization. While nanozymes primarily exhibit oxidoreductase or hydrolase activities, there is a need for ongoing exploration and development of other enzyme-mimicking activities to cater to the diverse requirements of tumor treatment. This will enable nanozymes to exhibit a wider range of functions.
(4) There is a need for more theoretical studies on multifunctional nanozymes in order to combine experimental and computational results and improve our understanding of their underlying principles. The catalytic mechanisms of these nanozymes remain unclear and require further investigation.

## 7. Conclusions and Prospects

TMO-based nanozymes exhibit excellent catalytic properties, unique photoelectric effects, and variable oxidation states due to the special electronic configuration of transition metal valence electrons. They have been widely developed and applied in biological fields. With the advancement of nanotechnology and a deeper understanding of catalytic mechanisms, the research on TMO-based nanozymes has been boosted. Furthermore, their higher stability and unique physicochemical properties make them highly suitable for biomedical applications. In this paper, we summarized the recent most commonly studied enzymatic activities of nanozymes and representative metallic oxide nanomaterials. Despite significant progress in the biomedical applications of TMO-based nanozymes, potential challenges, uncertainties regarding long-term biosafety, and key technical problems hinder its short-term clinical application.

In summary, the development of TMO-based nanozymes has made rapid progress and has shown broad application prospects in anti-tumor treatments. Although these therapies differ significantly from traditional treatments, their highly active performance and great potential make transition-metal-based nanozymes worthy of in-depth research. This review can provide a promising perspective and broaden the pathways for designing transition-metal-based nanozymes, which may benefit the rapid development of nanomedicine and biomedical materials in the future.

## Figures and Tables

**Table 1:** Typical examples of nanozymes with multienzymatic activities for enhancing tumor therapy.

Nanozyme	Transition Metal Ions	Enzymatic Activity	Applications	Target Tissue	Type of Study	Types of Cell	Types of Model	References
DMSN-Au-Fe_3_O_4_-CB839	Fe	GOx, POD	Fenton-like reactions, CDT	Subcutaneous tumor	in vitro/in vivo	4T1	BALB/c mice	[68]
rFeO_x_-HMSN	Fe	POD	Fenton-like reactions, *T*_2_-MRI	Subcutaneous tumor	in vitro/in vivo	4T1	Nude mice	[67]
UF@PPDF	Fe	POD, GSH-OXD	*T*_1_-weighted MRI, photothermal ferroptotic chemical synergistic cancer therapy	Subcutaneous tumor	in vitro/in vivo	HeLa	BALB/c mice	[66]
Ce6/MnO_2_@DPC NPs	Mn	CAT	Enhanced PDT	Subcutaneous tumor	in vitro/in vivo	U14	Nude mice	[76]
MS@MnO_2_	Mn	POD	MRI-monitored chemodynamic combination therapy	In situ implanted tumor	in vitro/in vivo	U87MG	Nude mice	[70]
UiO@Mn_3_O_4_@PAA	Mn	CAT, POD, GSH-OXD	Improve the efficacy of immunogenic RT by priming strong ICD	Subcutaneous tumor	in vitro/in vivo	4T1	BALB/c mice	[79]
Mn_3_O_4_ NPs	Mn	CAT, SOD	Treating ROS-related diseases	/	in vitro/in vivo	HeLa	Kunming mice	[78]
Cu_5.4_O USNPs	Cu	CAT, SOD, GSH-OXD	Against ROS-mediated damage	/	in vitro/in vivo	HEK293	BALB/c mice	[87]
Au/CeO_2_	Ce	OXD, POD	Superior antitumor effects both in vitro and in vivo	Subcutaneous tumor	in vitro/in vivo	SMMC-7721	BALB/c nude mice	[96]
Cu-CeO_2_ NPs	Ce	CAT, POD	Effective breast cancer therapy	Subcutaneous tumor	in vitro/in vivo	MDA-MB-231	Nude mice	[95]
Cu_2_O@Dex	Cu	POD	Enhanced CDT	Subcutaneous tumor	in vitro/in vivo	HepG2	ICR mice	[84]
Cu_2_O	Cu	POD, GSH-OXD	Fenton-like reaction, CDT	/	in vitro	AGS/MKN45	/	[88]
ZnMnFe_2_O_4_	Zn, Mn, Fe	POD,	Synergistic tumor cell diagnosis and ablation	Subcutaneous tumor	in vitro/in vivo	HepG2	Nude mice	[104]
MnMoO_x_	Mn, Mo	POD, GSH-OXD	Antitumor metalloimmunotherapy	Subcutaneous tumor	in vitro/in vivo	CT26	BALB/c	[103]
SnFe_2_O_4_	Sn, Fe	POD, GSH-OXD, CAT	Imaging-guided synergetic CDT/PTT/PDT.	Subcutaneous tumor	in vitro/in vivo	4T1	BALB/c nude	[111]
CuMnO_x_	Cu, Mn	POD, GSH-OXD, CAT, OXD	Photothermally enhanced multiple catalysis against tumor hypoxia	Subcutaneous tumor	in vitro/in vivo	HeLa	Kunming mice	[106]
